# Analysis of Multilevel Factors Mobilizing the Spectrum of Interorganizational Knowledge Sharing for Facilitating Digital Transformation at Scale: Qualitative Study

**DOI:** 10.2196/83345

**Published:** 2026-03-12

**Authors:** Hajar Mozaffar, Robin Williams, Kathrin Cresswell

**Affiliations:** 1 Business School, University of Edinburgh, 29 Buccleuch Place, Edinburgh, EH8 9LN, United Kingdom, 44 7846991188; 2 Institute for the Study of Science, Technology and Innovation, University of Edinburgh, Edinburgh, United Kingdom; 3 Usher Institute, University of Edinburgh, Edinburgh, United Kingdom

**Keywords:** digital transformation, health care sector, knowledge sharing, multilevel analysis, qualitative study

## Abstract

**Background:**

Interorganizational knowledge sharing is vital for scaling digital transformation efforts that span multiple organizations and system-wide change. However, existing frameworks provide limited insights into the cross-level dynamics that shape how learning ecosystems emerge, evolve, and operate across multiple organizations. This gap leaves practitioners without clear guidance on how multilevel contextual conditions and mechanisms interact to influence the development and sustainability of formal and informal knowledge-sharing relationships.

**Objective:**

This study aimed to examine how knowledge is orchestrated across organizations in the digital transformation of health care, identifying key factors that foster an evolving interorganizational learning ecosystem. We developed an integrative model that explains how these influences give rise to diverse modes of collaboration and partnership.

**Methods:**

We adopted a qualitative approach using a multilevel perspective to examine visions and experiences across individual, organizational, interorganizational, and sectoral levels. Drawing on a formative evaluation (2018‐2023) of England’s Global Digital Exemplar (GDE) program, we used multiple case studies and conducted interviews with experts both within and beyond organizational settings for data collection and adopted a grounded theory approach to analyze the data.

**Results:**

The study identified a set of interconnected factors operating at the macroenvironmental, interorganizational, organizational, and individual levels that influence how interorganizational relationships and partnerships are initiated, structured, and sustained. Macro-level influences included policy developments, program mandates, technology supplier strategies, and intermediary actions. Interorganizational mechanisms involved relational recognition, collective identity, governance structures, proximity, and coordination practices. Organizational factors included external search strategies, absorptive capacity, past collaboration experience, and internal knowledge routines. Individual-level mechanisms encompassed intrinsic and extrinsic motivations as well as personal inhibitors. Synthesizing these findings, we have proposed an integrative model that positions relationship type along a 2D spectrum (formal-informal, internal-external origins) and illustrates how different factors trigger, mandate, control, and enable the evolution of an interorganizational learning ecosystem.

**Conclusions:**

This study advances the theoretical understanding of learning ecosystems by explaining how multilevel contextual conditions activate mechanisms that give rise to diverse and evolving forms of interorganizational collaboration. Practically, we offer diagnostic and reflective tools that support policymakers and practitioners in assessing contextual conditions, selecting appropriate knowledge-sharing mechanisms, and monitoring how learning ecosystems develop over time. Our findings provide actionable guidance for designing and sustaining interorganizational learning systems capable of supporting digital transformation at scale.

## Introduction

Internationally, governments are enhancing and transforming their mode of operation, including processes, policies, and services, by adopting digital technologies, with the intention of improving service delivery; becoming more efficient and effective; and increasing transparency, interoperability, and public satisfaction [1,2]. The health care sector, in particular, is making substantial efforts to achieve digital transformation [3-7]. By digital transformation, we refer to extensive and complex initiatives, such as the implementation of hospital-wide or multisite systems, which bring about significant changes in organizational operations and workflows [7]. This involves advancing technological infrastructure and applications, as well as changing organizational practices, processes, structures, performance, and value creation mechanisms [8-11]. However, despite significant investment in digital health technologies by both government and supplier companies, the transformation of the health care sector lags behind many other sectors owing to the complexity of the health care system, lack of requisite know-how, and immaturity of the market [4,12].

The complexity of health care arises from the diverse and often fragmented pathways that patients must navigate in their journey back to health [13]. Health care systems also involve a multifaceted network of providers, which in some countries, such as the United Kingdom, Canada, Spain, and Singapore, operate under a single organizational structure yet remain functionally distributed and interdependent. Ongoing innovations introduce fundamentally new ways of delivering care, including greater integration across medical specialities, improved coordination between primary and secondary care, and closer alignment between health and social care services [14]. In addition, health care is shifting from predominantly reactive treatment models toward preventive and proactive approaches [15]. At the same time, expectations regarding patient safety and treatment effectiveness continue to rise. Collectively, these factors increase the demands on system performance and make effective coordination increasingly challenging [13].

Digital transformation requires new competencies and skills from those orchestrating the changes and those adopting new technologies for transformation [2]. These can be acquired through learning from other organizations that have already undergone similar digital change processes [10,13,16]. However, in digital transformation, experiences in one setting cannot be mechanistically applied in other contexts [12]. Effective change management, therefore, depends on *contextualized knowledge,* that is, adapting lessons from one organization so they fit the specific circumstances, needs, and constraints of the receiving setting [17].

Building on this, recent research shows that knowledge sharing is most effective when it occurs between organizations that are contextually similar, such as acute hospitals facing comparable pressures, infrastructure, and goals [18]. Such similarity allows organizations to more readily translate and adapt one another’s insights. To support this exchange, health care organizations engage in a range of interorganizational arrangements, evolving formal partnerships, exchanging formal codified knowledge (eg, through reports and blueprints), or sharing experience in informal ways (eg, site visits) [18-20]. These modes of engagement help organizations make sense of external lessons in ways that align with their own contexts, enabling more effective learning and application. In the rapidly growing field of digital transformation in the health care sector, policymakers promote and examine different interorganizational knowledge-sharing mechanisms [19,20], and of these, some may fail, while others may become sustainable or transform over time. Currently, however, the efforts in this area often proceed through trial and error, with limited systematic learning about what works and what does not. Emerging evidence suggests that while centralized, top-down approaches can be effective in certain circumstances, for example, by creating shared expectations or establishing a space for learning, they often do not translate into meaningful learning across diverse operational settings. In many cases, they fail to gain traction at the operational level [18].

Despite the recognized benefits of interorganizational knowledge sharing in digital transformation, there remains a notable gap in the literature concerning the specific factors that underpin effective interorganizational knowledge sharing, particularly in the health care sector, and how the factors mobilize a learning ecosystem that enables digitalization of the sector. In particular, while several frameworks offer valuable perspectives on digital transformation, they do not fully account for the *cross-level dynamics* that shape how learning ecosystems emerge, evolve, and function in practice, or *what forms* these ecosystems ultimately take. This theoretical gap has important practical implications: practitioners lack clear guidance on how multilevel contextual factors influence the development, effectiveness, and sustainability of formal and informal interorganizational learning relationships. Therefore, in this paper, we aim to explore and identify critical factors that shape the emergence and evolution of interorganizational learning ecosystems and to develop an integrative model that explains how these multilevel influences give rise to diverse modes of knowledge-sharing relationships during digital transformation.

To address these gaps, our study adopts a grounded, multilevel [21] analytical approach that enables us to trace how contextual conditions at different levels interact over time and across settings to shape processes of knowledge sharing. This approach allows us to identify recurrent patterns across diverse empirical materials and build an empirically grounded framework that explains how different forms of interorganizational learning arise and are sustained. The study therefore advances both the theoretical understanding of cross-level learning processes in digital transformation and the practical insights for cultivating effective interorganizational learning ecosystems in health care.

We draw on a recently completed formative evaluation (2018 to 2023) of a national digitally enabled transformation initiative in English provider organizations called *The Global Digital Exemplar (GDE) Program* to identify the range of factors and modes of interorganizational knowledge sharing as they unfold over time and multiple locations.

This work is timely, as it has the potential to inform policy and practice in relation to the growing investments being made in digital transformation of the health care sector not only in England but also in many other high- and middle-income countries. In doing so, the study provides a series of questions around factors and modes of knowledge sharing that contribute to enabling digital transformation, to be addressed by practitioners and policymakers.

## Methods

### Research Approach

In order to identify the factors at multiple levels of analysis contributing to interorganizational knowledge sharing, we adopted a longitudinal qualitative approach in the interpretive tradition [22], drawing on multiple case studies and conducting interviews with experts in the field within and beyond organizational settings.

### Research Setting

We studied a national digital transformation initiative in the English health care sector from 2017 to 2023, called the GDE Program. The GDE Program was a national investment in the transformation of a variety of care settings, including acute care, specialist care, mental health, and ambulance services of the National Health Service (NHS) in England, with a focus on digitally advanced sites to become international exemplars of excellence in order to provide learning for later organizations planning to go through digital transformation. In this program, provider organizations, henceforth referred to as GDEs, were paired with somewhat less mature fast follower (FF) provider organizations to promote learning across settings. GDEs and FFs captured the best practice models and lessons, including technologies, processes, and structures, in “blueprints,” which were aimed to be disseminated within and beyond the program to accelerate the spread of transformation knowledge. Shared learning in the program was also planned to be facilitated by learning networks launched in the first half of the program, bringing together GDE and FF team members with an interest in specific topics.

### Data Collection

#### Overview

We collected data using semistructured interviews, observations, and textual sources. These methods were used concurrently throughout the study period. Data collection was initiated through interviews, which were subsequently complemented by related observational and documentary data to deepen contextual understanding. In cases involving user groups, direct observations were conducted first and were followed by interviews to further explore emerging themes.

#### Sampling

We conducted in-depth case studies on a set of 12 provider organizations, carefully selected to ensure representation of both GDE and FF acute provider organizations, as well as mental health providers, reflecting the broader sample. The selection process aimed to include maximum variation, considering factors such as a range of different core electronic health record systems, geographical locations, and organization sizes.

Our participants included system vendors, national stakeholders such as program managers, policymakers, and digital health leaders who were responsible for setting digital strategy or who held formal roles such as Chief Clinical Information Officer, Chief Technology Officer, or equivalent positions. To ensure a diverse range of perspectives across the national digital health landscape, we purposively sampled interviewees operating within the NHS national program environment. Recruitment followed a systematic, multistage procedure. First, we identified potential participants through national program-level gatekeepers, including the senior responsible owner overseeing the implementation of the wider program, who provided access to individuals directly engaged in strategic and operational decision-making. Second, we used our existing professional networks to identify individuals holding formal digital leadership roles within NHS organizations who were actively involved in digital transformation efforts. Finally, we used a snowballing approach by asking each participant to recommend others, particularly those who might hold alternative perspectives, to further broaden the diversity of views included in the study.

#### Semistructured Interviews

We conducted interviews with 228 recognized experts from NHS England professional domains, policymakers, and suppliers, who were involved in the design, launch, monitoring, evaluation, or running of the GDE Program at some point in time. Rather than being formally pilot tested, the guides were iteratively tailored to the expertise and role of each interviewee to ensure relevance and depth of discussion. The questions aimed to explore the evolving nature of knowledge-sharing modes, relationship types and their features, and factors influencing interorganizational knowledge exchange. Data collection was conducted in both face-to-face and online settings. Interviews typically ranged from 30 to 90 min in duration, with an average length of approximately 1 hour. Data were audio-recorded where permission was granted. For nonrecorded interviews, extensive notes were taken. Participants were offered the opportunity to review the final study report, and several provided feedback and comments at that stage. Data collection continued until thematic saturation was reached, at which point no new concepts or insights emerged from the data.

#### Observations

To complement data collected through interviews, observations of meetings related to GDE Program activities and user group events focused on particular digital technologies being implemented within participating organizations. These observations enabled us to understand how different stakeholder groups engaged with the technologies, addressed challenges, and coordinated implementation work. In addition, we observed 3 national conferences centered on the digitalization of the NHS, where program leaders and adopter sites shared their experiences, lessons learned, and implementation strategies. During the observations, we captured the setting layout, the way participants moved and interacted, the actors, insights into processes and discussion content, and insights into interaction outcomes.

#### Textual Data

Additionally, to complement the observations and interviews, we collected relevant documents, such as business plans, blueprints, lessons learned documents, and implementation documents, to gain insights into the type of knowledge being shared and relevant processes. To understand broader system-level discourse, we supplemented this with data from online forums, such as NHS Futures and the Digital Health Network, focusing on what knowledge was shared, how it was communicated, and the forms of support or collaboration that emerged across the wider digital health community.

### Data Analysis

Our data analysis integrated well-established approaches, including longitudinal case analysis, grounded theory development, and content analysis. The process was iterative, requiring continuous movement between the data and the evolving theoretical framework [23]. However, for clarity, we present our analysis as a structured sequence of 5 main stages. In stage 1, the lead researcher, HM, utilized NVivo 14 (Lumivero) to organize and integrate all data sources and conducted initial open coding to capture the breadth of stakeholder views and experiences. This stage generated a large pool of provisional codes across interviews, observations, and documents.

In stage 2, we adopted a focused coding strategy to move from initial descriptive codes to analytically meaningful first-order codes. Guided by the principles of parsimony, conceptual salience, and recurrence across data types, we treated sentences and paragraphs as coding units [24] and read the full dataset iteratively, including catalogs and supporting materials. We assigned labels using in vivo codes where appropriate or concise descriptive terms and then grouped related segments into coherent conceptual categories [25]. Importantly, our intention at this stage was not to catalog every detail within such a large dataset but to distil patterns that were recurrent, theoretically relevant, and directly related to our research questions. This analytic focus explains why the final set of first-order codes is relatively concise (n=33). To ensure robustness, coding decisions were discussed within the research team at multiple points, allowing for refinement of categories, challenge of interpretations, and agreement on the conceptual boundaries of each code. Throughout this process, we examined perspectives at multiple analytic levels, including individual, adopter organization, government, and technology supplier, as well as across different stages of digital transformation (procurement, adoption, and use). This enabled us to generate a focused yet comprehensive set of first-order codes that meaningfully represented mechanisms shaping knowledge sharing across organizations.

In stage 3, we identified links among ﬁrst-order codes to form the emerging second-order themes [24]. This involved discussing disconfirming evidence within the extended research team to minimize the risk of researcher bias.

Stage 4 involved linking second-order themes to form aggregate dimensions [24,26], associated with our research question. These 3 stages (2, 3, and 4) formed our data structure, as presented in Table 1. The Results section begins with explaining this data structure in detail.

**Table 1.** Categorization of key factors contributing to knowledge sharing.

Aggregate and second-order themes	First-order codes
Macroenvironmental factors	
Policy developments	Formation of GDE^a^/FF^b^ pairings as a prerequisite of GDE funding Blueprinting activities mandated by the GDE Program Digital Academy supporting skill development/cohort/cadre of digital transformation leaders Wachter Review leading to the development and advancement of new roles such as CCIO^c^/CNIO^d^ Formation of the Digital Health Network as an NHS^e^-organized community
Technology supplier strategies	Co-ordinating technology-specific user groups Planning pilot site visits Suppliers diversifying the technology market
Intermediary actions	Independent technology consultants moving across settings Formation of communities of interest by adopter organizations
Interorganizational factors	
Relational recognition	Development of collective identity (eg, GDE status or Digital Aspirant status) Naming organizations as GDEs (indicating their digital maturity) as opposed to FFs (indicating them as followers)
Structural governance mechanisms	Time- and resource-bound contracts versus ad hoc interactions Need to exchange knowledge in a structured repository (eg, blueprints) versus unstructured sharing (eg, email or site visit)
Interorganizational proximity	Organizations with closer geographical proximity forming formal GDE/FF partnerships Personnel from geographically closer organizations communicating and moving between sites Cost sharing or synergy seeking
Organizational factors	
External search strategies	Identifying a solution to a certain problem Identifying who has implemented particular technologies Setting future digital transformation strategies
Absorptive capacity	Internal knowledge acquisition strategies Time allocation to participate in community or networking events
Past experience	Managing knowledge requests from external organizations based on past experience
Individual factors	
Intrinsic motivations	Reputational and professional status
Extrinsic motivations	Creation of communities of shared interests
Inhibitors	Personal inhibitors
Spectrum of interorganizational relationships and partnerships	
Origins of partnerships/relationships	Policy (eg, formal agreements with GDE/FF sites) Independent groups of enthusiasts (eg, Digital Health Network) Provider organizations (eg, periodic or ad-hoc meetings, trainings, and events) Suppliers (eg, technology user groups and pilot site visits)
Governing structures and durations	Formal contractual agreements Flexible periodic engagement Informal need/opportunity basis

a GDE: Global Digital Exemplar.

b FF: fast follower.

c CCIO: Chief Clinical Information Officer.

d CNIO: Chief Nursing Information Officer.

e NHS: National Health Service.

In stage 5, after identifying the aggregate dimensions in stage 4, we noted strong parallels between our grounded analysis and context-mechanism-outcome (CMO) [27] configurations described in realist literature, that is, the taxonomy of multilevel factors incorporated context and mechanisms, and shaped the spectrum of interorganizational relationships and partnerships (outcomes). Building on the similarities, we propose a framework that shows the relationship between the aggregate dimensions. We structured the reporting of this study in accordance with the COREQ (Consolidated Criteria for Reporting Qualitative Research) guidelines to enhance rigor, transparency, and completeness [28].

### Ethical Considerations

We obtained institutional review board approval from the University of Edinburgh Research Ethics Committee (27.11.2017). Informed consent was obtained from all participants prior to their involvement in the study, and all data collected were anonymized to ensure confidentiality and protect participant and organizational privacy. Participants were not compensated for their time.

## Results

### Overview

Overall, our dataset consisted of 228 interviews; observations of 113 national strategic meetings, user groups, and conferences; analysis of 277 documents; and desk research into online platforms of knowledge-sharing settings such as forums and webinars. Table 2 presents the interviewee characteristics. We present the findings under 2 key headings: multilevel factors contributing to the sharing process and the spectrum of interorganizational relationships and partnerships. Subsequently, informed by the CMO perspective, we present an integrative view of how the factors across 4 levels—macroenvironmental, interorganizational, organizational, and individual—influence knowledge-sharing modes. The factors identified in each category are not exhaustive; however, they highlight some of the most recurrent factors identified in our data within each category.

**Table 2.** Interviewee characteristics.

Characteristic	Value, n (%)
In-depth case study sites (12 sites; n=178)	
CCIO^a^	34 (15)
Program manager	29 (13)
CIO^b^	27 (12)
Nursing roles	15 (6)
Digital director/lead	12 (5)
CNIO^c^	11 (5)
Project manager	11 (5)
Pharmacist	7 (3)
Other IT-related roles	20 (9)
Clinical transformation lead	6 (3)
Benefit realization lead	6 (3)
Wider data collection (n=50)	
Policy	44 (19)
Industry	3 (1)
Others	3 (1)

a CCIO: Chief Clinical Information Officer.

b CIO: Chief Information Officer.

c CNIO: Chief Nursing Information Officer.

### Taxonomy of Multilevel Factors Contributing to the Interorganizational Knowledge-Sharing Process

#### Macroenvironmental Level

Our study identified 3 key groups of macroenvironmental factors that influenced knowledge sharing across the settings.

First, *policy developments,* such as government funding and training programs, were seen to have a profound impact in terms of knowledge sharing across different sites. For example, the development of the NHS Digital Academy, which was a close collaboration between several universities and the NHS, supported the skills development of health professionals and decision makers to become digital transformation leaders. In parallel with this, new Digital Health Network communities formed, and these together brought over 5500 NHS digital health leaders to share knowledge and collaborate through online communities, regional and annual events, and webinars. Another example is the Wachter Review conducted between 2015 and 2016, led by an external digital health expert (from the United States), which reviewed the digitalization of the NHS and led to several recommendations that were put into practice, such as the development and advancement of new roles (eg, Chief Clinical Information Officer and Chief Nursing Information Officer) as mediator roles between digital transformation decision makers, front line health care staff, and middle management. In these 3 examples, the macroenvironment was an enabler of transformation and a promoter of knowledge sharing across organizations.

*And then out of that, so I go to digitalhealth.net events so, like, the summer schools and the leaderships, something that they do, I go to that…I’m part of the One HealthTech, I don’t know if you’ve heard of that, so it’s like a volunteer organization for women in health tech. So I represent [site_A]. So there are some other forums where I’ve access to other people and you tend to meet…there’s lots of networking at those events…. So I get lots of emails from people, like, the CNIOs and people from other trusts [hospitals] just asking questions and I have their contact so I can email them. So there’s quite a lot of informal, once you’ve met somebody, that, kind of, networking, you just…you know that they’re doing a certain thing if you want to know about that, you can contact them.*

[Participant from Site_I]

Despite their enabling roles, the policy development factors may sometimes hinder effective collaboration across organizational settings. For instance, some sites involved in GDE/FF partnerships highlighted that their pairings had been established on the basis of policy criteria, often under compressed timeframes, sometimes resulting in a lack of effective engagement between the partners.

Second, *technology supplier strategies* affected the types of relationships being formed and the collaborations between different organizations. For instance, in the GDE Program, supplier-coordinated networking activities, such as user groups, pilot site visits, and connections of key individuals across organizations, were common. However, many of these supplier-organized initiatives, while being beneficial in initiating conversations, in particular, with international partners, only allowed for supplier-set goals.

*When you go on a site visit, you’re chaperoned around, no one lets you talk to anyone. Even when you go on the PACS visits, we were definitely stuck in a room and not allowed out.*

[Participant from Site_AG7]

In the majority of cases, interviewees highlighted that supplier-arranged learning opportunities drive adopter organizations in particular directions, such as buying particular digital technologies, rather than allowing for open conversations.

*…the problem with that is that [user group] if you take supplier_name, for example, it’s always going to be about supplier_name, well, you should do it this way with this supplier_name product, it’s not always the right choice.*

[Participant from Site_C]

However, over time, the nature of supplier-organized groups has evolved, becoming increasingly user-driven and confident. For example, users became more active in certain user groups regarding electronic prescribing systems, collaborating to hold suppliers more accountable. Similarly, in a different user group setting, the supplier encouraged their adopters to commit to share learning, reinforcing the shift toward more reciprocal and empowered user-supplier relationships.

Furthermore, other supplier strategies, such as diversifying the market by, for instance, offering best-of-breed solutions on one end of the spectrum as opposed to mega-suites at the other end of the spectrum, had an indirect effect on how and which adopter organizations communicated.

Finally, *intermediaries* had a noticeable influence in orchestrating the sharing of knowledge, particularly where formal policy-developed structures or supplier strategies were deemed to be limited in effectiveness. There are different types of intermediaries, ranging from individuals, such as consultants and technical experts, who facilitate sharing by providing specialized professional services to organizations, to public and private entities that provide technology brokerage, such as suppliers and individual consultants.

*…Lessons Learned paper that I had, I was given that on day one, when I started, and I went through that myself, and since then, there really hasn’t been much engagement at all. Now we have had a specialist that worked for Site_Name on their EDRMS solution…We’ve put 15 days into that, so obviously quite a lot, it’s £750 a day…He brought a lot of experience to the table from what Site_Name did right, and what they did wrong. So, effectively, he was our pseudo blueprint.*

[Participant from Site_M]

User-driven communities organized around a particular interest, such as a specific type of technology, are also effective intermediaries, which facilitate learning and cross-organizational collaborations.

*…we decided, we need a user group, and we need to engage with other users, so that we’ve got a collective voice for the suppliers. So, we established the national user_group_name, and our CIO was the chair of that…And we also established something called the user_group2_name, which was for the more techies, about how to manage upgrades, how to do certain things in the system, so it’s like a knowledge sharing, question and answer thing. And we asked every trust [hospital] to chip in a nominal amount of fund, we’re talking about 200 quid…*

[Participant from Site_E]

In general, macroenvironmental factors impacted interorganizational knowledge sharing both directly and indirectly. In direct terms, they mandated (eg, blueprinting activities in the GDE Program) and structured relationships (eg, GDE and FF relationships formed between digitally advanced organizations and less digitally advanced organizations to share experiences). In indirect terms, they influenced the ability and motivation of organizations to form relationships for knowledge-sharing purposes (eg, user communities).

#### Interorganizational Level

Our analysis highlighted 3 types of interorganizational factors influencing knowledge sharing across the settings.

First, we identified that *relational factors,* including the formation of collective identity, power relationships, and trust between organizations, directly affected knowledge-sharing practices. The development of collective identity expanded communication across organizations of similar titles. For instance, we observed that different types of interorganizational collaboration events and meetings were set up (either by adopter organizations or by the NHS), with reference to the status of the organization being a GDE hospital. In such events (eg, the GDE network 2019 event in London or a meeting between Site_D and Site_F), the collective identity was used as an anchor for communication and collaboration.

In terms of power relationships, for instance, our analysis showed how the labeling of organizations as being digitally advanced (GDEs) or being follower organizations (FFs) impacted the dynamics of sharing. While in some instances, the relationship created a means for GDEs to share their advanced competencies with their FFs, in other instances, it hindered sharing, as some FFs did not see themselves as lagging behind in competence and capability. Some FFs, therefore, did not aspire to learn from their GDE peers. This formal power relationship, which differentiated hospitals in their digital status, could also, in some instances, get in the way of sharing knowledge across 2 digitally advanced sites.

*It’s a very strange place the…well, you’d have to say that some trusts [hospitals] that aren’t GDEs would feel that they’re much more digitally mature than the GDEs. So, whilst the GDE programme singled out a number of trusts [hospitals] for these programmes, you almost then say to those trusts [hospitals], when everybody’s looking in, they’re saying, we’re better than them, or actually, we can do a better job than them. So, you don’t tend to get that learning going across, I suppose, I don’t know, how much learning do you get between [Site_A] and (Site_B]. Or, if someone develops some software in [Site_A], would [Site_B] take it? You might find that [Site_B] said, well do you know what, we’ve got something better, or actually if we did x, y and z, we’d…so it’s that competition, in what is supposed to be a national health service, there is that reluctance to adopt other people’s ideas and technology.*

[Participant from Site_B]

Second, our study pointed to the importance of *structural relationship governance mechanisms* between organizations in enabling or hindering the knowledge-sharing process. For instance, the formal partnerships were planned based on clear strategic organizational needs by strategic decision makers. They prescribed how knowledge exchange was to take place and how the communications were organized. This afforded very different knowledge-sharing practices to informal relationships, which were formed on an ad-hoc basis and were based on particular local needs, the experiences of each organization, aspects that were beneficial, and organizational availability. Additionally, the structure of the relationship, including how it was governed and what routines and processes were involved in knowledge interactions, had a direct effect on different aspects of knowledge sharing. In terms of governance, for instance, whether the relationship is an informal relationship that is established and runs based on the availability of different organizations, or whether it is based on some type of contractual association with a defined scope and defined roles, affects the type of knowledge being exchanged. This includes, for example, structured documents with codified knowledge on how to implement a functionality versus discussions on solving a particular problem using system configuration and workarounds during a networking event, the direction of knowledge flow (eg, from a GDE to an FF or multidirectional across multiple organizations), the frequency of interactions (eg, a specific number of site visits in a given period of time vs ad-hoc site visits based on requirements), and the extent and longevity of sharing.

Finally, *interorganizational proximity* influenced knowledge sharing across organizations. Proximity has different aspects, such as physical proximity or the degree of technological or strategic similarity. For instance, organizations with a closer geographical proximity either formed formal GDE/FF partnerships or had personnel communicate and move between sites readily in informal ways to exchange knowledge. Another example was the procurement and implementation of similar technologies by different hospitals, which led to the development of relationships/partnerships, with motivations such as cost-sharing or synergy-seeking.

*Then I think because at the same time [Site_C] were going through and had decided to also take [technology_Name] as its electronic patient record system, they were our formal fast follower, and so it enabled us to work with them. But in fact we had always agreed that we would provide wider support to other hospitals also considering or implementing [technology_name]. So as well as [Site_C], we were providing support to [Site_D], [Site_E] and [Site_F].*

[Participant from Site_A]

#### Organizational Level

We observed that 3 organizational factors had a direct influence on the sharing of knowledge.

First, an organization’s *agenda for searching for external knowledge* was an important factor. Some organizations only looked for external knowledge when a particular need arose, such as the need for the implementation of a new piece of technology, while others broadened external knowledge search mechanisms to identify unknown or future strategic opportunities.

*I think the other thing we sort of did over the summer, was have a look at all of the GDE blueprints. I know that we’re a GDE, so I suppose the assumption is that, because we’re a GDE, we’ve done everything, but I’m a great believe in learning from somebody else. So, we kind of did a trawl of all of the blueprints that have been published to date, to see whether or not there was anything we’re not taking use of as much as somebody else is, and really starting to think about, what’s our next cycle of digital activity? And you can kind of see there, that pretty much the first two columns says, oh, we’re probably doing about 85 per cent of everything that’s out there, but the final column says, there are a few options. And I think, in particular for us, it’s interesting that we’re doing webinars for our staff, but not for our patients.*

[Participant from Site_L]

Second, an organization’s *absorptive capacity* played an important role in enabling sharing across organizations. Absorptive capacity refers to an organization’s ability to acquire, assimilate, and utilize external knowledge, information, and innovations. For instance, some organizations had implemented internal processes and routines to facilitate learning and sharing, enabling them to acquire insights from other organizations and manage external knowledge-sharing demands. Conversely, in cases where less time was dedicated to absorbing and utilizing external knowledge, such as during the procurement of new digital technologies, these organizations gained limited benefits from the knowledge held by others, which sometimes led to experiencing challenges previously faced by other organizations. Some interviewees highlighted that their organization had established a routine for them to allocate time for periodic conversations with other organizations, enabling structured opportunities for knowledge exchange. By introducing these routines, organizations can foster a culture of continuous learning, gain insights from the experiences of peers, and adopt the best practices in digital transformation. This approach not only accelerates their own digital initiatives but also helps them navigate challenges more effectively, ultimately strengthening their overall organizational capabilities.

*…pretty much every month the CIOs or heads of IT delivery […] have opportunity to meet each other …to actually talk to each other. And that makes the world of difference…the other week, [name] went along and came back with a small list of this is what I’ve got from [org_name_A], this is what I’ve got from [org_name_B], could you have a chat with this person please…I just ring them up and say, yeah, you wanted a chat, this is what it’s about and vice versa…*

[Participant from Site_G]

Finally, an organization’s *past experiences* in terms of identifying, building, and maintaining relationships had a noticeable impact on the formation of its knowledge-sharing practices. For instance, organizations that were more experienced in managing and coordinating relationships established clear processes for managing the requests from external organizations asking for their knowledge and experiences.

*They were asked not to make direct contact with [site_B] staff, they had to go through a specific office and up to monitor, well not monitor but manage and control the contacts. Which worked fine actually because they were as I said, they replied very quickly when we begin a request, which was usually by email and we got through that pretty quickly. They wanted to avoid loads of people making contact with loads of [site_B] staff and disrupting them unnecessarily.*

[Participant from Site_C]

#### Individual/Interpersonal Level

We found 3 individual factors impacting formal and informal knowledge sharing across organizations.

First, we observed that individuals are driven to share knowledge by *intrinsic motivations*, specifically their self-interest. Our observations revealed that participants exhibit a heightened willingness to share knowledge when they perceive tangible benefits arising from such collaborative endeavors. Among these motivating factors, the prospect of bolstering one’s reputation and professional status emerged as a significant catalyst for active involvement in knowledge-sharing communities.

Furthermore, the desire to gain profound insights into alternative perspectives and diverse experiences emerged as another compelling factor propelling knowledge sharing. Participants acknowledged the richness that emerges from exposure to a variety of viewpoints, and they mentioned actively seeking to broaden their understanding by tapping into the collective wisdom of others.

*so that’s been really helpful, to have that outside perspective from someone else in a different Trust [hospital] doing something that’s similar but having a different viewpoint. As I find the people are fantastic, so much knowledge, so much experience, but I appreciate we’re all at the same Trust [hospital] so we’ve got almost like the same vision, and sometimes it’s just nice to see someone else’s perspective who’s not part of this Trust [hospital].*

[Participant from Site_E]

Second, beyond intrinsic motivations, *extrinsic factors* rooted in altruism played a pivotal role in fostering the sharing of knowledge across organizational boundaries. The altruistic dimension of knowledge sharing is particularly evident in instances where individuals perceive that their contributions go beyond personal gain and actively contribute to the construction or sustenance of communities of shared interests. Participants recognized that by sharing their expertise, insights, and experiences, they are instrumental in nurturing a collaborative environment where collective learning and problem-solving thrive. This communal aspect became a source of motivation, as actors derived satisfaction from the sense of building and sustaining a network of like-minded individuals. Furthermore, the fostering of feelings of companionship and camaraderie emerged as a powerful intrinsic motivator for knowledge sharing. Participants found fulfillment in being part of a community where the exchange of knowledge is not solely transactional but is imbued with a sense of shared purpose and mutual support.

*we’ve now set up a GDE group with everyone that looks after benefits for their Trust [hospital], and we’re using it as a sharing platform to help each other…*

[Participant from Site_E]

While our study did not aim to identify barriers, in the case of individual factors, we encountered individual inhibiting factors that introduced complexities for the sharing of knowledge that necessitated careful consideration. Three notable aspects emerged as noteworthy impediments: the allocation of time to engage in sharing activities, the concept of knowledge-based psychological ownership, and the nuanced emotional responses individuals exhibited when navigating the uncertainties inherent in interorganizational settings. The time commitment required for participation in knowledge-sharing activities proved to be a substantial factor. Individuals, often operating within demanding professional environments, found themselves grappling with the challenge of allocating sufficient time to contribute meaningfully to knowledge-sharing initiatives.

*The is a CSO network but unfortunately, we don’t meet terribly often. And actually, most of us are just too busy…but in the end the problem is it doesn’t get enough time, I think. I am still a full time clinician, so I still have to do a very much full time on-call job.*

[Participant from Site_G]

Some individuals were possessive of their knowledge, struggling with what to share openly versus what to protect for their organization’s benefit. This sense of ownership created barriers to the free flow of insights. Additionally, concerns about partners’ behaviors affected the willingness to share, introducing a complex balance between trust and caution. Apprehensions about defining knowledge boundaries (what to disclose and what to withhold) further highlighted the nuanced nature of interorganizational knowledge sharing. These individual perspectives and emotional responses shaped knowledge transfer dynamics, underscoring the need for a deeper understanding to foster effective collaboration.

### Linkages Across Levels in Activating Knowledge-Sharing Modes

The factors identified in our study do not operate in isolation; rather, elements across macroenvironmental, interorganizational, organizational, and individual levels are highly interconnected and often mutually reinforcing in shaping knowledge-sharing relationships. For example, national policy developments, such as the Wachter Review, not only stimulate the creation of new digital leadership roles (eg, Chief Clinical Information Officer and Chief Nursing Information Officer) but also contribute to the emergence of a digitally skilled cadre through initiatives like the NHS Digital Academy, thereby improving the absorptive capacity of organizations and their ability to engage meaningfully in communities of practice. Similarly, program-level structures, such as mandated GDE/FF pairings and blueprinting requirements, interact with relational dynamics, including the formation of collective identities (eg, GDE status) and the development of trust-based recognition between partner sites, which in turn facilitate voluntary knowledge exchange beyond formal contractual obligations. Technology supplier strategies also intersect with organizational search behaviors: suppliers’ diversification of the market can drive adopter organizations to seek external knowledge (eg, identifying who has implemented specific technologies), often aided by intermediary actors or technology-specific user groups that bridge otherwise disconnected sites. Geographical proximity further amplifies these interactions, as staff from nearby organizations are more likely to visit one another, participate in shared communities of interest, or move across settings, thereby strengthening informal knowledge flows that complement formal governance mechanisms. Finally, individual-level motivations, such as professional reputation, intrinsic interest, and past experience in handling knowledge requests, shape how actors engage with these structures, while personal inhibitors can constrain knowledge sharing even when enabling conditions are otherwise present. Together, these interconnected elements illustrate how multilevel factors combine to activate and shape the modes underpinning knowledge sharing across the health system.

### Spectrum of Interorganizational Relationships and Partnerships

Together, the above interconnected conditions contributed to a spectrum of interorganizational relationships and partnerships—what we refer to as modes of knowledge sharing—that emerged from multiple origins and were shaped by varying degrees of structure and formality. These mechanisms ranged from highly orchestrated arrangements to more flexible, organically developed connections. Across this spectrum, organizations used diverse tools and practices for knowledge sharing as described below.

The *origins* of relationships ranged from internal (individual or communal within and across transforming organizations) to external (beyond the transforming organizations). In the case of internal origins, sometimes provider organizations collaborated on a local needs basis (eg, site visits or phone calls between individuals) or more strategically entered into intentional cultivation of networks and communities in response to collective needs or to achieve collective strategic benefits. For instance, when multiple organizations needed particular functionalities from the same vendor, organic and spontaneous collaborations between 2 or more user organizations helped to create a collective force to exert leverage over technology vendors or to collaborate and form a technological solution that benefited multiple organizations.

*And then on going forward it was about where we had joint requirements for development such as around various different areas such as out-patients or theatres. We’d try and come up with a joint specification so that [supplier_name] only had to develop once.*

[Participant from Site_D]

In the case of external entities initiating relationships, in some instances, interorganizational relationships were initiated by formal mandates from the government. For instance, in our study, 19 strategic pairings were formed between GDEs and FFs. These partnerships were based on a range of factors such as common core technologies and local strategic groupings, for instance, between organizations that had a particular electronic patient record system or between nationally coordinated collaborations of health care organizations and local authorities.

Technology suppliers were also initiators of particular types of relationships around specific technologies, both nationally and internationally.

*...there’s now quite good, not only UK, but a wider European group forum. Plus, we have regular conversations with Melbourne. Pre-[technology_name], our pediatricians used to go back and forth with Melbourne. Melbourne physicians used to come here, our guys used to go there, so there’s a very strong relationship with Melbourne. They have [technology_name]. It’s a bit like an onion, you know, layers and layers of different communities that have formed very nicely.*

[Participant from Site_A]

The wide spectrum of interorganizational relationships had different *governing structures and durations*. For instance, government-initiated partnerships could range from national conferences or conference series (short-term, flexible periodic engagements) to fixed-term/long-term contracts between different provider organizations (eg, GDE/FF partnerships). These contracts mandated a number of aspects, such as the type of sharing (documentation), the interval for sharing, and what is to be shared. Despite investments into these formal means of knowledge sharing, many of the initiatives did not reach their anticipated longer-term benefits, despite being easy to measure, and their purposes changed over time. One example is blueprints, which were mandated by the GDE Program and were designed as tools for sharing the knowledge needed to implement specific models of change to be adopted by the wider ecosystem. While they offered codified knowledge about new process designs, they did not provide the means to implement certain technologies in the new context, as the implementation process involved a range of tacit knowledge that was not included in the blueprints. Many provider organizations used blueprints as a tool to identify which organizations and which actors were sources of particular knowledge in order to establish informal contact and to allow for the sharing of knowledge beyond codified documents.

We also noted that not all formal initiatives proved to be self-sustaining over time. For instance, GDE Program managers launched a number of learning networks, many of which were terminated after the end of national funding. Only some networks driven by professional agendas and interests (eg, the Hospital Electronic Prescribing Medicines Administration Network) sustained and transformed into national communities of practice after the completion of the initial funding period. Collaboration and sharing of knowledge in these communities were not based on policy-mandated contracts, and instead, knowledge sharing was orchestrated through collaboration of provider organizations as they captured local and collective benefits from contributing to the community.

### Integrating Influencing Factors Into the Spectrum of Interorganizational Relationships

**Figure 1.**
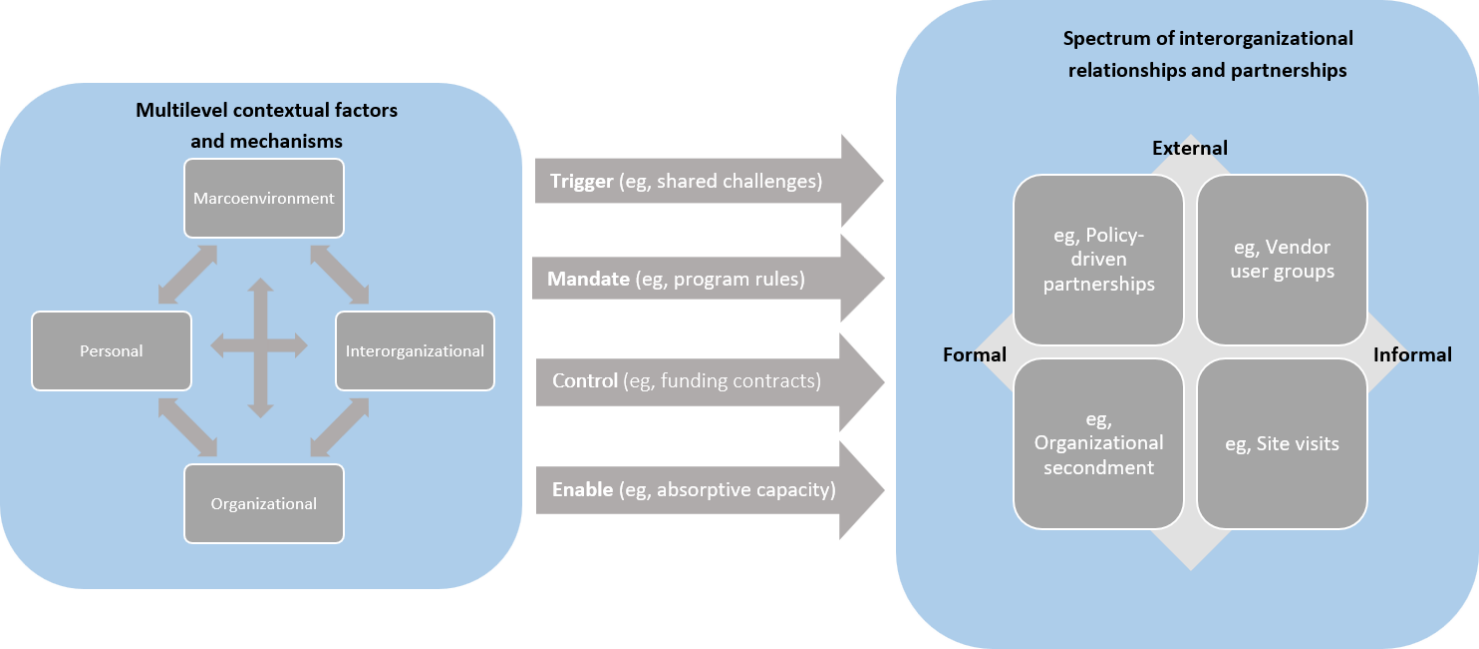
Integrative model of interorganizational learning ecosystems in health care digital transformation.

Building on these findings, we developed an integrative model (Figure 1) that brings together the multilevel factors (context and mechanisms on the left-hand side) identified in our analysis with the resulting spectrum of interorganizational relationships and partnerships (outcomes on the right-hand side). The model illustrates how different contextual conditions and mechanisms combine to produce a diverse array of knowledge-sharing relationships, which vary not only in formality but also in their roots and modes of initiation. To capture this variation, we conceptualized a 2D spectrum: the x-axis represents the degree to which a relationship is formal or informal, while the y-axis indicates whether its origin is internal (arising from organizational strategies, leadership roles, or intrasystem initiatives) or external (emerging from policy directives, supplier strategies, intermediary actions, or professional networks). Importantly, the left and right sides of this spectrum are connected through a range of influences, such as triggering, mandating, controlling, and enabling, which shape, prompt, or support interorganizational knowledge sharing across different settings. By positioning relationship types within this matrix, the model demonstrates how combinations of enabling factors give rise to different patterns of collaboration and partnership across the ecosystem, offering a coherent way to understand the diversity and dynamics of interorganizational knowledge-sharing arrangements.

## Discussion

### Principal Findings

Our findings show that effective interorganizational knowledge sharing is shaped by different configurations of factors linking across the levels of a system. At the macro level, policy stability, national investment, and the presence of formal digital transformation programs created perceived legitimacy, shared purpose, and a sense of mandate among organizations, resulting in higher engagement in knowledge-sharing activities and more formalized collaborations. At the interorganizational level, networks characterized by trust, prior relationships, and aligned implementation timelines enabled modes of reciprocal exchange, openness, and problem-solving, leading to sustained cross-site learning, joint solution development, and more mature partnerships. In contrast, competitive procurement pressures or misaligned incentives triggered protective behaviors, producing fragmented or transactional exchanges. These examples of different configurations illustrate how and why knowledge-sharing modes vary in effectiveness and evolve over time.

### From Knowledge Sharing to Learning Ecosystems: A Theoretical Contribution

In this section, we compare our empirical findings with the broader literature on knowledge sharing across organizations and present how our findings contribute to the development of the concept of evolving learning ecosystems.

Combining our findings with the extant literature that addresses *macroenvironmental factors* on interorganizational knowledge sharing, we identified a wide range of macroenvironmental factors, including policy developments, intellectual property regimes [29], technological sophistication levels [30], technology suppliers and their strategies [31], diverse markets [32], and other external agencies (eg, universities, civil society groups, third-party firms that have close connections with the health care system, etc), which can trigger, mandate, control, or enable interorganizational knowledge sharing. Policy-related factors can act as “windows of opportunity” [29] that enable or mandate the formation of relationships for sharing specific types of knowledge [33]. However, the outcome of using the mechanisms mandated or enabled by policies is dependent on how knowledge-sharing stakeholders exploit these opportunities [18]. For instance, when the government creates formal partnerships and mandates the sharing of documents in a repository accessible to a wide range of organizations, the scale of sharing may be large, but the scope and depth of sharing may be limited. Other external entities, such as suppliers (through their strategies) and intermediaries, also shape the types of relationships being formed and the collaborations that may take place between different organizations [30]. Typically, relationships formed by suppliers are less formal, sometimes ongoing, and sometimes on an ad-hoc basis. A common finding in the GDE Program was supplier-coordinated networking activities such as user groups, pilot site visits, and connections of key individuals across organizations. Intermediaries may offer formal partnerships by providing specialized professional services to organizations and facilitating sharing between them [34,35] or providing technology brokerage activities [36]. Intermediaries may also enable the formation of communities of interest, where the relationship is less formal. In general, the macroenvironmental factors impact interorganizational knowledge sharing both directly and indirectly. In direct terms, they can mandate (eg, blueprinting activities in the GDE Program [18]) and structure relationships (eg, GDE and FF relationships formed between more digitally advanced organizations and less digitally advanced organizations to share experiences [37]). In indirect terms, they can influence the ability and motivation of organizations to form relationships, for example, by identifying who holds what knowledge about a particular type of technology.

*Interorganizational factors*, including relational factors [38], structural governance mechanisms, and interorganizational proximity [38], have a profound impact on shaping the early stages of relationship/partnership building. Our research shows that by building a collective identity that involves the development of a common objective or a mutual win and involves shared rules and regulations in terms of both formal [39] and informal [40] relationships, knowledge sharing can be encouraged. These can be achieved through building interorganizational teams or communities of interest that invest in modes of knowledge exchange and integration in order to solve a problem or overcome a common challenge [41-43]. Additionally, structural governance mechanisms (eg, whether they are time-bound or not, or whether they are periodic or run on an ad-hoc basis) also affect the type of relationship and the direction of knowledge flow. Furthermore, organizational proximity plays an important role in the formation of both formal and informal sharing. The extant literature highlights various aspects of organizational proximity, including cultural closeness [44-46], similarity in technological artifacts in use [47], alignment in strategic direction [48], and geographical proximity, and these facilitate smoother mobility of personnel, socialization, in-person exchange, and informal ties [36,49].

In terms of *organizational factors*, our study highlights the importance of knowledge search strategies by organizations, absorptive capacity, and past knowledge-sharing experiences in triggering and enabling sharing, as well as a wide spectrum of interorganizational knowledge-sharing modes. The extant literature has focused on the importance of absorptive capacity, which refers to the ability to recognize the value of knowledge being shared by both the donor and receiver organizations and the ability to utilize it [50]. The absorptive capacity is influenced by an organization’s past experiences of identifying and building relationships [51], ability to integrate knowledge [52], and capability to retain knowledge [53]. We also found that as organizations gain experience in sharing and managing knowledge demands, they become more capable of managing and coordinating relationships as well as organizing the learning process. Organizations may use tools, such as IT-enabled systems, to eliminate barriers of communication and thereby heighten the level of knowledge sharing.

In relation to *individual and interpersonal factors*, we highlight the role of intrinsic and extrinsic motivations in encouraging knowledge sharing and the personal inhibitors that hinder the sharing process. Research has revealed a range of explanations for why individuals contribute to knowledge sharing in formal and informal settings. Many explanations point to intrinsic (self-interest) and extrinsic (altruistic) motivations [54]. Such motivations include self-benefits received from participation in knowledge-sharing communities and settings, such as access to expert advice [55], insights into others’ beliefs and opinions, enhanced reputation and professional status [56,57], networking [58], and improved self-image and confidence [59]. Benefits to others include a desire for building or sustaining communities of interest [59,60], feelings of companionship [57], and reciprocity [61]. These motivations may vary in terms of nature and depth in formal and informal knowledge-sharing relationships. Bateman et al [62] highlighted that an individual’s commitment to the organization they belong to, as well as the community, has a significant effect on participating in knowledge sharing. In this way, individuals may believe that they benefit from knowledge-sharing relationships [63] in both formal and informal settings, or they may feel obliged to pay back to existing relationships [56,62]. While the literature highlights the role of motivations in facilitating knowledge sharing, it also identifies factors that may hinder the transfer process. These include the time required for sharing activities [59] and the feeling of a strong psychological ownership over knowledge [63]. Emotional attachment to the organization may also discourage individuals from sharing certain knowledge, as it leads them to prioritize the organization’s interests [64], influencing their decisions on what knowledge to protect and how to control its release [65].

Our study contributes to the literature on digital transformation and interorganizational learning by advancing the understanding of how learning ecosystems emerge, evolve, and take shape across multiple levels of the health care system. Rather than treating macroenvironmental, organizational, interorganizational, and individual factors as discrete categories, we demonstrate that they operate as dynamically interconnected influences that jointly shape the evolution of knowledge-sharing relationships. Through our multilevel analysis, we show how contextual conditions, such as national policy directives, program mandates, supplier strategies, and organizational digital maturity, activate mechanisms, including identity formation, absorptive capacity, motivations, and relational recognition. These mechanisms, in turn, produce a spectrum of relationship types that vary in formality and origin, ranging from informal peer interactions to mandated partnerships.

By integrating these insights, our work conceptualizes the *learning ecosystem* as an evolving sociotechnical system in which relationships and knowledge flows continually adapt in response to shifting contextual pressures and emerging opportunities. We demonstrate that learning ecosystems do not develop through linear or centrally orchestrated processes; instead, they emerge through recursive interactions across levels, where changes in one domain, such as policy initiatives or new digital leadership roles, can reconfigure organizational strategies, reshape interorganizational networks, and influence individual patterns of knowledge sharing. The integrative model we propose brings these dynamics together by illustrating how multilevel factors (contexts and mechanisms) give rise to diverse modes of interorganizational collaboration (outcomes), and how triggers, mandates, controls, and enablers collectively shape the system’s trajectory over time. This contribution extends existing frameworks on interorganizational knowledge sharing by offering a holistic explanation of how learning ecosystems are formed, how they function, and why they evolve differently across settings. In doing so, it provides theoretical clarity for understanding and supporting the development of interorganizational learning ecosystems capable of enabling digital transformation at scale.

### Implications for Practice

This study offers important implications and insights that can be used in enabling and supporting the digital transformation process in the health care sector. Drawing on our findings, we have developed a structured set of questions designed to help policymakers and practitioners diagnose the conditions shaping knowledge sharing and monitor how these conditions evolve over time. These questions focus on the macroenvironmental, interorganizational, organizational, and individual factors that influence the emergence and functioning of learning ecosystems. We present these questions in Table 3. These questions serve as both a diagnostic aid (helping stakeholders assess what they are trying to achieve and the contextual factors shaping these efforts) and a monitoring aid (helping them evaluate how learning conditions and relationships develop over time).

To address macroenvironmental questions, policymakers and practitioners must examine public policies that facilitate or hinder the formation of formal and informal partnerships, as well as the role of external agencies in enabling knowledge sharing. Additionally, it is important to identify external factors, such as competition and cooperation. This requires assessing whether policymakers or other external entities demand the sharing of specific types of knowledge and whether industry characteristics, such as the levels of technological complexity and market maturity, affect partnership and community-building. To answer interorganizational questions, policymakers and practitioners should explore structural governance mechanisms such as community-based orchestration, contracts, defined processes, and routines. They must also examine relational and cognitive governance factors, including trust and power dynamics. Exploring organizational questions requires assessing the capabilities and experiences of donor and recipient organizations in forming and managing partnerships or participating in communities. This includes their ability to manage alliances, learn from and apply external knowledge, and facilitate knowledge sharing. Additionally, behavioral aspects, such as organizational motivations, incentives for knowledge sharing, and resistance to new knowledge, must be considered, along with organizational characteristics like the credibility of the donor and the receiver’s flexibility to adapt to change. Finally, addressing individual-level questions requires examining both intrinsic and extrinsic factors influencing knowledge-sharing behavior.

**Table 3.** Diagnostic and monitoring questions for assessing multilevel factors influencing knowledge sharing.

Factor level	Questions
Macrocontext factors	What formal external factors (eg, regulations, policies, technology, and market) affect the motivation to form formal partnerships for knowledge sharing? What external factors affect the motivation to share knowledge without the existence of formal partnerships? Is there an agency external to knowledge-sharing organizations that structures, incentivizes, and monitors knowledge sharing? What is the role of strategic mandates?
Interorganizational factors	How do the interactive dynamics of the relationship (ie, power relations, structural governance and mechanisms, social ties and trust, and risk) influence knowledge sharing? How does proximity (geographical, cultural, and technological) affect knowledge sharing? What are the relationship-building factors (ie, collective identity, mutual goals, and joint rules) that affect knowledge sharing?
Organizational factors	How do organizational characteristics affect knowledge sharing? How do routines, processes, and coordination mechanisms affect knowledge sharing? What is necessary for an organization to share/receive external knowledge?
Individual factors	What motivates individuals to share knowledge? What are the individual factors that prevent the sharing of knowledge?

Building on this diagnostic foundation, we offer a second set of questions that guide stakeholders in identifying and selecting appropriate modes and mechanisms of knowledge sharing for digital transformation initiatives. Rather than prescribing a fixed sequence of steps, these questions encourage reflection on the purpose of different knowledge-sharing modes, their temporality, the coordination techniques they require, and the tools that support them. These questions are presented in Table 4. Stakeholders can populate them with context-specific examples and options relevant to their initiatives. Table 4 includes 3 illustrative columns to demonstrate how different knowledge-sharing modes may be examined and compared.

**Table 4.** Reflective questions for selecting and assessing knowledge-sharing mechanisms in digital transformation initiatives.

Question	Option 1	Option 2	Option 3
What are the different options for formal/informal interorganizational knowledge sharing?	Blueprinting	Online user communities	Site visits
What purposes does it serve?	To share how a particular technology has been configured and implemented	To identify solutions to specific problems/needs; To co-innovate	To observe real-world technology use and challenges; To validate assumptions
What is the duration?	Project based	Continuous; Need basis	One-off
Who are the initiators of the modes of sharing?	Government	Individuals	Organizations; Technology suppliers
What are the management/coordination techniques?	Controlled by funding contacts	Orchestrated by community members	Managed by organizations on a need basis
What infrastructure and tools are used to facilitate knowledge sharing?	Documentation tool and database	Forums	In-person visits

Consistent with existing literature emphasizing the importance of relational maturity and temporality in knowledge-sharing processes [45,52,66,67], we propose that the questions in both tables should be revisited and iteratively assessed over time. Together, Tables3  4 provide a structured yet flexible resource to support the design, implementation, and continual refinement of interorganizational learning within digital transformation programs.

By addressing these 2 sets of questions, this study provides policy and practice tools and guidelines to enhance and accelerate the digital transformation of the health care sector. First, it identifies where and how governments and other intermediaries can support interorganizational knowledge sharing. Second, it highlights the formal and informal modes and internal organizational and individual factors that organizations can leverage as they navigate the transformation journey.

### Limitations and Future Research

While this study advances the understanding of how learning ecosystems emerge and evolve across multiple levels, several limitations create opportunities for future research. First, our analysis focused predominantly on macroenvironmental and interorganizational factors, reflecting the design of the national digital transformation program and the nature of the available data. As a result, less attention was given to micro-level organizational routines and individual behaviors that may shape, or are shaped by, the learning ecosystem. Second, although our multicase design enabled rich comparative analysis across organizations, the scope of the study was limited primarily to hospital settings within a national health care system. Learning ecosystems may function differently across other care settings, sectors, or international contexts with different governance, market, or cultural dynamics. These limitations offer a valuable foundation for theoretical propositions that can guide future research aimed at advancing, refining, and testing the model developed in this study.

To guide such research, we propose a set of theoretically informed propositions. These propositions posit that cross-level contextual alignment is central to effective ecosystem formation; mechanisms, such as identity and relational recognition, significantly influence knowledge flows; the balance of formal and informal relationships affects ecosystem adaptability; and intermediaries are essential brokers in sustaining learning systems. Moreover, we propose that external influences shape early ecosystem trajectories, internal factors become more influential as ecosystems mature, learning outcomes vary with specific factors, and organizational- and individual-level factors warrant a deeper empirical investigation. Together, these propositions establish a clear agenda for advancing the theory on the emergence, evolution, and functioning of interorganizational learning ecosystems in digital transformation contexts.

### Conclusion

Digital transformation has presented a significant challenge in the health care sector, which requires knowledge, experience, and expertise for organizational gains and new value creation. Interorganizational knowledge sharing can be seen as an enabler, supporter, and accelerator in assisting digital transformation at scale. Policymakers and organizations must proactively address the internal and external factors that impact knowledge sharing to harness its full potential in driving digital transformation. Understanding and evaluating approaches to creating a learning ecosystem is far from a trivial task and requires multilevel inquiries that follow actions within and beyond the organization in understanding this complex phenomenon.

Our framework illustrates how multilevel influencing factors shape a spectrum of interorganizational relationships and partnerships. The framework explores the dynamic nature of factors over time and therefore goes beyond shedding light on snapshots of the knowledge-sharing process by offering an evolutionary view of the process and its outcomes. By proposing this framework, we do not suggest that the domain of interorganizational knowledge sharing can be simply reduced to the aforementioned dimensions. Moreover, we do not propose this as a simple mechanistic account that could decouple different variables for the testing of modes of sharing and factors. Instead, we present a set of dimensions that are essential to explore in order to theorize a conceptual account of the learning ecosystem. We introduce this not just as an abstract framework but as a practical tool to support reflection and guide action for the various actors involved in shaping and sustaining it.

## Abbreviations

CMO: context-mechanism-outcome
COREQ: Consolidated Criteria for Reporting Qualitative Research
FF: fast follower
GDE: Global Digital Exemplar
NHS: National Health Service
